# Inhalational Heroin Use and Leukoencephalopathy: A Case Report

**DOI:** 10.7759/cureus.42323

**Published:** 2023-07-23

**Authors:** Brandon W Knopp, Hannah Z Weiss, Michele Retrouvey, George Luck

**Affiliations:** 1 Endocrinology, Florida Atlantic University Charles E. Schmidt College of Medicine, Boca Raton, USA; 2 Medical Education, Florida Atlantic University Charles E. Schmidt College of Medicine, Boca Raton, USA; 3 Radiology, Florida Atlantic University Charles E. Schmidt College of Medicine, Boca Raton, USA; 4 Integrated Medical Science, Florida Atlantic University Charles E. Schmidt College of Medicine, Boca Raton, USA

**Keywords:** neurology, neuroimaging, critical emergency medicine, heroin use, heroin-induced leukoencephalopathy

## Abstract

Heroin-induced leukoencephalopathy (HLE) is a rare condition with acute and chronic outcomes ranging from mild neurological symptoms to severe neurological deficits and death. HLE is caused by cerebral white matter damage secondary to exposure to toxic agents such as chemotherapeutic drugs, environmental toxins, and drugs of abuse. Here, we present the case of a 20-year-old woman with a past medical history significant for bipolar disorder and opioid use who presented to the emergency department with ataxia, involuntary movements, and altered mental status secondary to inhalational heroin use. The patient presented with symptoms including agitation, tremors, speech difficulty, confusion, memory loss, and weakness. Magnetic resonance imaging (MRI) showed diffuse cerebral atrophy and electroencephalography (EEG) was significant for cerebral dysfunction in the left hemisphere and diffuse encephalopathy. The patient was treated with intravenous (IV) steroids, vitamins, and fluids but failed to show improvement. She was subsequently discharged to hospice 17 days after admission. There are few reported cases of toxic leukoencephalopathy due to heroin inhalation. The patient’s young age and presentation following one month of abstinence are particularly unique as she suffered an acute decompensation with severe, lasting neurological deficits. This case highlights a potential presentation of HLE and seeks to increase clinical recognition in patients with a recent history of substance use and unexplained neurological symptoms.

## Introduction

Heroin-induced leukoencephalopathy (HLE) is a rare condition with acute and chronic outcomes ranging from mild neurological symptoms to severe neurological deficits and death [1-3]. Common symptoms include cerebellar ataxia and dysarthria, while generalized motor impairments and altered mental status may present in more severe cases [3,4]. The timeframe of symptom development is highly variable as some patients develop symptoms progressively during a prolonged stretch of substance use. In contrast, others can acutely develop symptoms soon after beginning substance use. The factors predisposing to an acute or chronic presentation, as well as symptom severity, are unknown. As only around 200 cases have been reported to date, further reporting is required to better understand this disease [1,2].

We present a case of a 20-year-old woman who developed severe neurological damage due to HLE following one to two years of inhalational heroin use. This case is unique because the rapid onset of debilitating symptoms occurred after one month of abstinence from heroin use. It highlights an atypical presentation of HLE and informs providers of a potential presentation of this rare condition.

## Case presentation

A 20-year-old female with a past medical history significant for bipolar disorder, herpes simplex virus, and inhalational heroin use was brought to the emergency department (ED) by her father for symptoms including altered mentation, involuntary movements, and ataxia. The patient reported that she had minor memory issues and occasional forgetfulness over the past few months. She had no other reported symptoms until four days before admission. At this time, she experienced a rapid memory decline, dysmetria while eating, and minor behavioral changes. Upon presentation, she quickly deteriorated with an inability to walk, decreased attentiveness, speech difficulty, dysphagia, and a mixture of choreoathetotic and hemiballismic movements. Additional symptoms included confusion, agitation, tremors, weakness, body rigidity, diffuse back pain, and bladder incontinence. She was partially responsive at this time. She reported that her last known use of heroin was one month ago and she had been on a buprenorphine/naloxone combination treatment since.

The vitals were largely unremarkable. She was afebrile, had an elevated respiratory rate of 26, and was mildly tachycardic with a heart rate of 105 beats per minute. She was cooperative with a Glasgow Coma Score of 15 and was oriented to person, place, and time. Pupils were equal, round, and reactive to light, and both eyes exhibited abnormal extraocular motion. Her speech was delayed and slurred. She exhibited diaphoresis, intermittent agitation, chorea-type movements, diffuse hyperreflexia, tremor, a positive Babinski sign bilaterally, abnormal coordination with dysmetria, an inability to ambulate, spasticity, and bilaterally decreased breath sounds. She had no abdominal masses, hepatosplenomegaly, or rashes. She displayed normal strength and range of motion.

**Figure 1:**
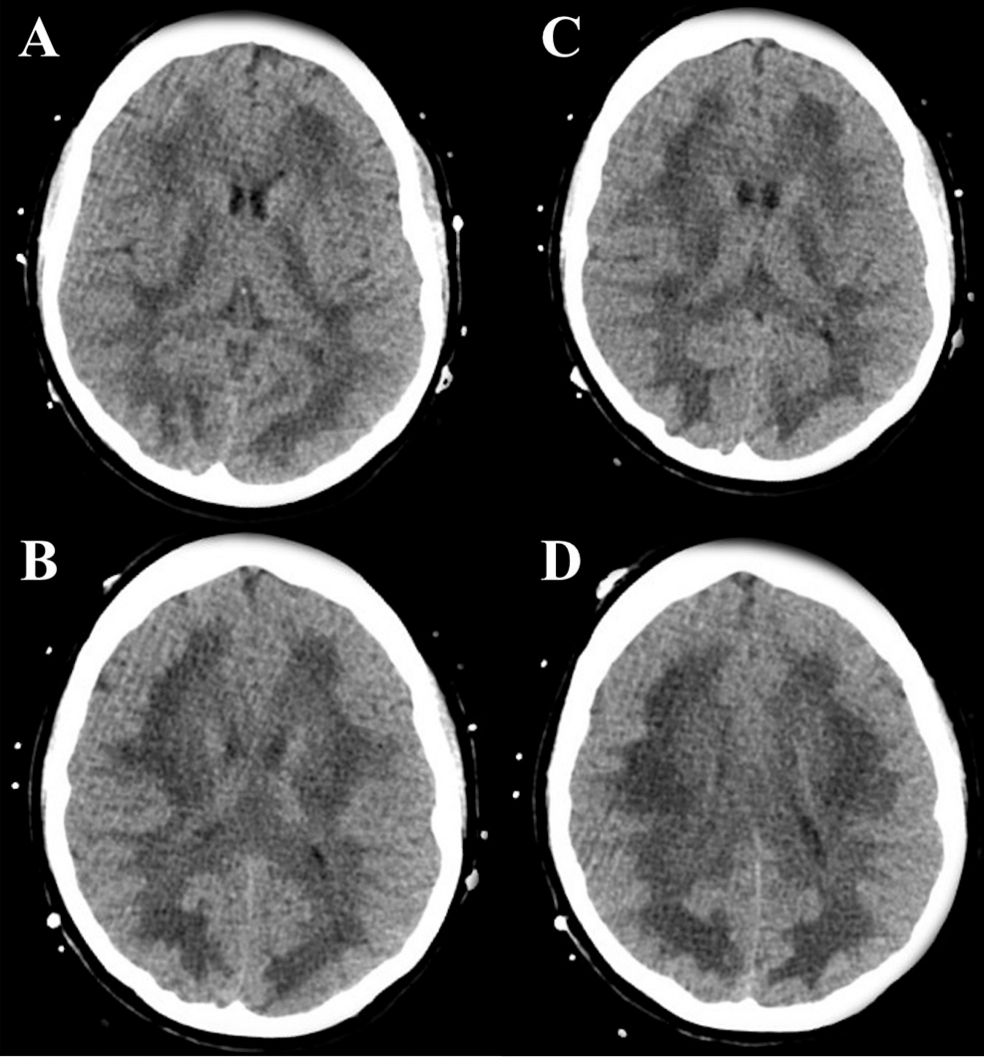
Representative images of the CT head without contrast obtained on admission. There is diffuse symmetric hypodensity throughout the white matter, more pronounced at the corona radiata, without involvement of the gray matter. Panels are arranged from most caudal (A) to most cranial (D).

Significant laboratory results are shown in Table 1. Urine drug screening was positive for benzodiazepines and oxycodone. An initial computerized tomography (CT) scan of the head without contrast was obtained and showed confluent white matter hypodensity (Figure 1).

**Table 1:** Significant Laboratory Results

	Laboratory Results	Reference Values
Glucose	133 mg/dL	< 140 mg/dL
Chloride	107 mEq/L	95–105 mEq/L
Creatinine Kinase	265 U/L	10–70 U/L
Calcium	10.5 mg/dL	8.4–10.2 mg/dL
Bicarbonate (HCO_3_)	19 mEq/L	22–28 mEq/L
Absolute Neutrophil Count	7,620/mm^3^	2,500-8,000/mm^3^
White Blood Cell Count	12.83/mm^3^	4500–11,000/mm^3^
Red Blood Cell Count	3.53 million/mm^3^	3.5–5.5 million/mm^3^
Hemoglobin	10.8 g/dL	12.0–16.0 g/dL
Hematocrit	30.7%	36%–46%

**Figure 2:**
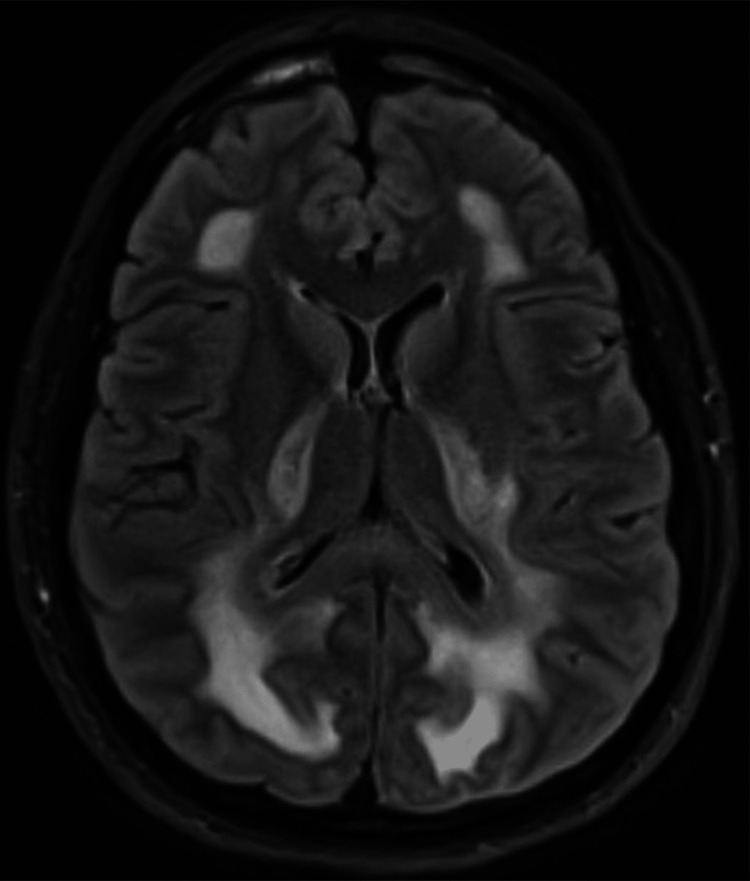
FLAIR sequence from the patient’s initial MRI demonstrates abnormal hyperintense signal in the white matter of the frontal lobes, in the posterior limb of the internal capsule, and extending into the white matter of the parietal and occipital lobes. There is sparing of the subcortical U-fibers and the deep gray nuclei. FLAIR: fluid-attenuated inversion recovery

A contrast-enhanced magnetic resonance imaging (MRI) scan demonstrated a confluent, symmetric non-enhancing transverse relaxation time (T2)-weighted fluid-attenuated inversion recovery (FLAIR) signal hyperintensity involving the supratentorial white matter (Figure 2).

**Figure 3:**
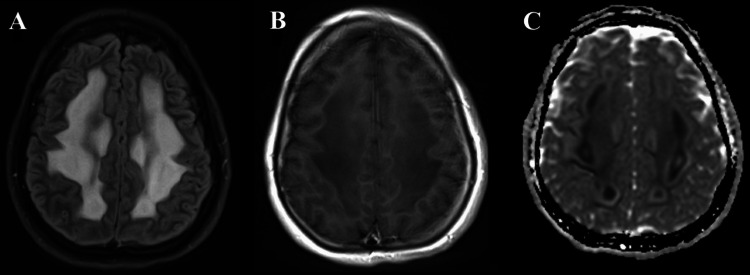
MRI images from the same level demonstrate a high signal on FLAIR (A), without enhancement on the post-contrast T1 images (B). Restricted diffusion is noted throughout the affected areas, as seen on the ADC (C). FLAIR: fluid-attenuated inversion recovery; ADC: apparent diffusion coefficient

In the areas of high signal on FLAIR (Figure 3A), there was no evidence of enhancement on the post-contrast longitudinal relaxation time (T1) images (Figure 3B), but extensive restricted diffusion was present (Figure 3C).

**Figure 4:**
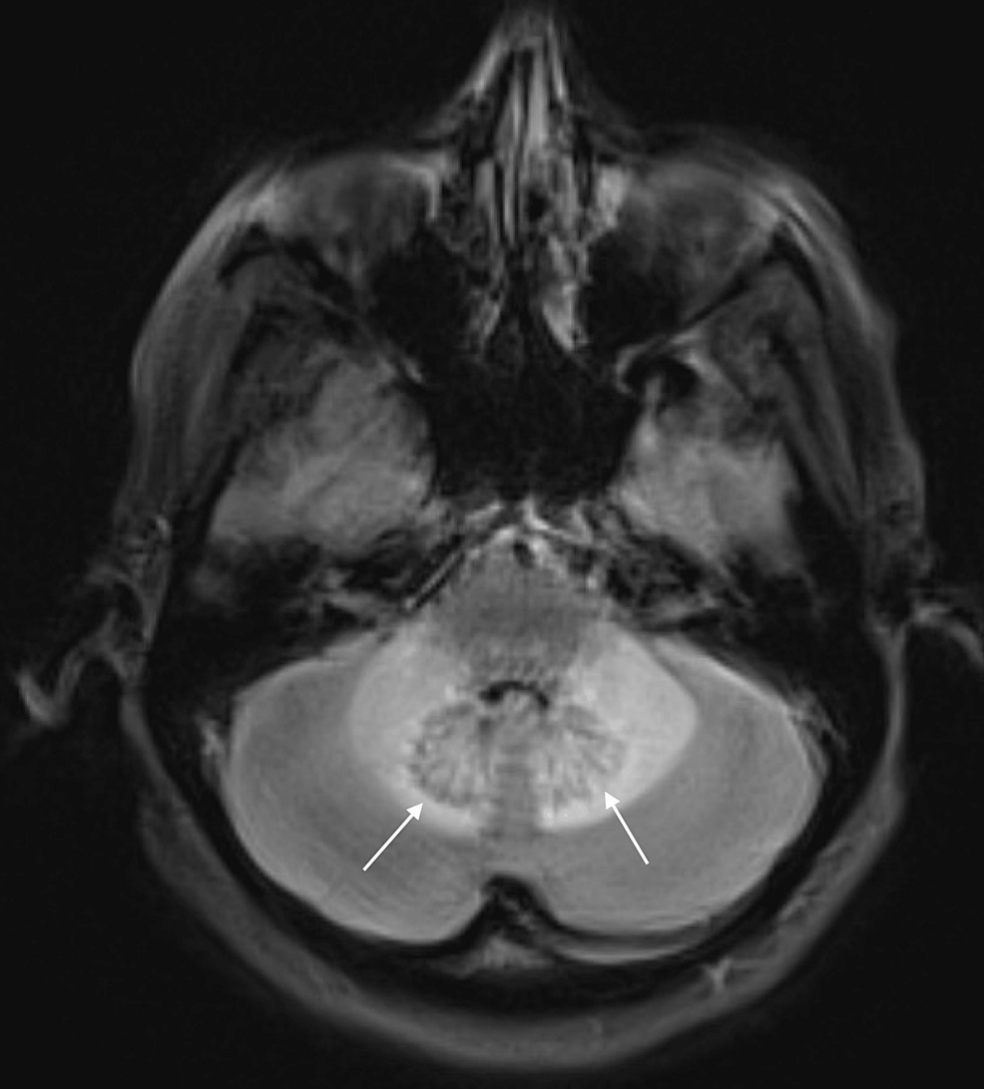
T2 FLAIR images demonstrate symmetrical involvement of the cerebellar white matter and middle cerebellar peduncles in a “butterfly wing” pattern (Arrows). FLAIR: fluid-attenuated inversion recovery

The cerebellar white matter and middle cerebellar peduncles were involved in a symmetric “butterfly wing” pattern (Figure 4), consistent with heroin inhalation toxicity.

During the MRI, the patient developed a fever (102°F rectal), tachycardia, and rigidity. She was subsequently admitted to the intensive care unit (ICU) with suspected neuroleptic malignant syndrome. Initial treatment included two liters of low-flow oxygen, intravenous (IV) fluids, and an IV vitamin infusion. After five unsuccessful lumbar puncture attempts, empiric antibiotic coverage with acyclovir, ceftriaxone, and vancomycin was begun over meningitis concerns. A repeat lumbar puncture two days later was noninfectious, resulting in the discontinuation of acyclovir and vancomycin. During the first five days of admission, she was also administered IV methylprednisolone, which was subsequently tapered off and discontinued as no clinical improvement was appreciated. A repeat MRI had no significant changes from imaging taken on hospital day one and diffuse, severe white matter injury characteristic of toxic or metabolic leukoencephalopathy. Electroencephalography (EEG) showed cerebral dysfunction in the left hemisphere and severe diffuse encephalopathy.

The patient was intubated on hospital day four for acute hypoxic respiratory failure requiring mechanical ventilation with chest X-ray findings indicative of pulmonary edema, which resolved on subsequent imaging. The patient had a percutaneous endoscopic gastrostomy (PEG) tube placed 11 days after admission. Despite improvement in pulmonary edema, the patient remained intubated and had a tracheostomy placed 16 days after the initial presentation. At this time, the patient displayed severe agitation, involuntary movements, upper extremity rigidity, inability to follow commands, unresponsiveness to verbal stimuli, mild responsiveness to painful stimuli with a moan or grimace, and extreme obtundation. The patient was ultimately stabilized and transferred to hospice care 17 days after admission.

## Discussion

HLE is a rare condition presenting with potentially severe neurological deficits which can lead to permanent disability and death [5]. Though heroin and its metabolites can cross the blood-brain barrier, the precise mechanisms and substances underlying neurologic injury due to HLE are unknown [1,2,6]. Determining the underlying cause of neurologic damage in HLE is further complicated by the varying clinical presentations seen with HLE [2]. The present case posed challenges in determining pathophysiology due to the atypical symptom progression a month after cessation of heroin use. The patient experienced forgetfulness, mild behavioral changes, and dysmetria for three to four days before rapidly decompensating with debilitating symptoms leading to significant disability and inability to perform basic activities of daily living. She recovered from acute hypoxic respiratory failure and was extubated during her hospitalization but never completely recovered, prompting a hospice discharge.

The rapid symptom onset, particularly in the setting of a young patient with only one to two years of inhalational heroin use, is remarkable in this case. This is unique among reported cases of HLE due to the acute onset of debilitating symptoms in a young patient after a month of abstinence from heroin. This presentation after a month of reported abstinence suggests that a cumulative effect of neurotoxic substance intake results in progressive damage. However, cases of acute neurological injury after the first usage have been reported [7]. The mode of heroin use likely plays a role and different routes of administration (inhalational, IV injection, snorting, and transconjunctival) have differing rates of leukoencephalopathy. However, precise rates are not well-described [6,7]. Likewise, risk factors for severe presentation are not well-understood. Reasonable hypotheses for contributing risk factors include routes of administration with a higher neuropenetrance, length of use, neurosusceptibilty due to preexisting conditions, and stage of brain development. However, more data is required to substantiate these inferences. Factors that may have predisposed this patient to rapid deterioration may include her diagnosis of bipolar disorder, heroin use at a young age before neurological maturity, and the inhalational route of heroin use.

Early recognition and management are important considerations for patient care. HLE presentation varies between cases with neurological symptoms that can have an insidious onset over weeks or a rapid onset in days. This presentation can be easy to miss and requires a high index of clinical suspicion to diagnose and treat patients in a timely manner.

## Conclusions

HLE is a potentially debilitating condition with unknown pathophysiology and varying symptomatology characterized by diffuse, symmetrical white matter changes on brain imaging. HLE is difficult to diagnose, but with a high index of clinical suspicion, more patients with HLE can be diagnosed early and treated before irreversible neurological damage occurs. This report seeks to describe a possible presentation of HLE and increase clinical recognition in patients with a recent history of substance use and unexplained neurological symptoms.
